# (Eco)Toxicity of E-Waste: Current Methods, Challenges, and Research Priorities

**DOI:** 10.3390/toxics13121048

**Published:** 2025-12-03

**Authors:** Diogo A. Ferreira-Filipe, Andrew S. Hursthouse, Armando C. Duarte, Teresa Rocha-Santos, Ana L. Patrício Silva

**Affiliations:** 1 Centre for Environmental and Marine Studies (CESAM) & Department of Chemistry, University of Aveiro, 3810-193 Aveiro, Portugal; aduarte@ua.pt (A.C.D.); ter.alex@ua.pt (T.R.-S.); 2 School of Computing, Engineering & Physical Sciences, University of the West of Scotland, Paisley PA1 2BE, UK; andrew.hursthouse@uws.ac.uk; 3 Centre for Environmental and Marine Studies (CESAM) & Department of Biology, University of Aveiro, 3810-193 Aveiro, Portugal; ana.luisa.silva@ua.pt

**Keywords:** metals, e-microplastics, persistent organic pollutants, ecotoxicity, key organisms

## Abstract

The rapid growth in manufacturing and use of electrical and electronic equipment has led to unprecedented volumes of poorly managed e-waste, posing serious ecological risks. Although data on individual chemical substances in e-waste are available, evidence of ecotoxicity from actual e-waste materials remains scattered. This review consolidates organism-level ecotoxicity data on real e-waste samples (mixed fractions, fragments, leachates) and samples collected near e-waste facilities (soil, sediments, dust, water) across aquatic and terrestrial environments. It critically examines how methodological approaches influence reported outcomes and outlines research priorities. In aquatic environments, toxic responses vary with increased amounts of toxicants (dissolved metals, particles from dismantling operations) that mobilise to surface waters, while hydrophobic organic compounds cause sublethal behavioural and genotoxic effects. The few studies on terrestrial environments show impaired invertebrate growth and reproduction, along with changes in soil and “plastisphere” microbiota. However, tested concentrations, material complexity, and incomplete reporting of exposure chemistry, among other factors, limit the environmental relevance and comparability of the data. Uniformised procedures, combined with thorough chemical characterisation, environmentally realistic conditions, and cross-system bioassays (including different exposure routes and cumulative assessments), may provide mechanistic insights into e-waste toxicity, supporting evidence-based risk management strategies while contributing towards the development and validation of robust new approach methodologies (NAMs).

## 1. Introduction

The increasing use of electrical and electronic equipment (EEE) in modern societies has driven intense competition to develop innovative products that support daily life and business. This rapid technological progress, shorter product lifespans, and expanded market reach have significantly increased electrical and electronic equipment waste (also known as e-waste) [1]. E-waste is composed of diverse materials, including metals, plastics, and additives, and this complexity of materials imposes great challenges in terms of waste management. The variety of form factors, components, and materials requires specialised separation, sorting, and treatment options, limit the economic attractiveness of e-waste recycling. For example, while metal recovery from e-waste is well investigated and economically viable [2], the treatment of e-waste plastics and glass, often contaminated with persistent organic pollutants, does not benefit from the same economic incentive. Consequently, a large fraction of e-waste is exported to regions with less stringent environmental and industrial regulations, where it is processed through informal recycling, landfilling, or incineration of low-value components such as plastics, potentially resulting in uncontrolled emissions of hazardous chemical substances [3].

Previous reviews have addressed the ecotoxicity of individual constituents of e-waste, such as e-plastics (e.g., Karbalaei et al., 2018 [4]; Prata, 2024 [5]), metals (e.g., Leyssens et al., 2017 [6]), and flame retardants (e.g., Xiong et al., 2019 [7]), as well as the human health impacts of e-waste exposure (Ankit et al., 2021 [8], Eze and Vinken, 2024 [9]). A systematic review has also been released [10], providing an overview of e-waste toxicity research up to 2022, primarily from a bibliometric perspective. Isolated assessments may not accurately reflect the toxicity of e-waste in real-world scenarios, where organisms are exposed to a complex mixture of pollutants capable of inducing more severe adverse effects through additive or synergistic interactions [11,12]. Despite growing recognition of the environmental and public health implications of improper e-waste management, knowledge gaps persist regarding the ecotoxicity of actual e-waste materials and contaminated environmental matrices under realistic exposure conditions.

This review provides a unique focus on studies investigating the environmental and human health effects of samples from e-waste per se, e-waste facilities, and adjacent environments—including whole discarded devices, disassembled/shredded devices, leachates/extracts, particulate matter, soil, sediments, and water. Its novelty lies in the emphasis on environmentally relevant samples and exposure scenarios, alongside a critical evaluation of the methodologies and experimental approaches used across recent research (2019 onwards). To conduct this review, Scopus and Google Scholar were used, with a search strategy involving the terms “e-waste”, “WEEE” (waste EEE) or “electronic waste” in conjunction with “toxicity” or “ecotoxicity” within the “Article Title, Abstract, Keywords” fields, from 2019 to September 2025. The results were then refined to include only (eco)toxicity studies that employed e-waste (whole) samples, e-waste-derived toxicants/leachates, samples collected from/or nearby e-waste processing sites, or samples collected from exposed populations.

## 2. E-Waste Ecotoxicity Assessment: Laboratory to Field Approaches

Despite the growing environmental concerns associated with e-waste pollution, the number of (eco)toxicity studies conducted in the past 6 years using e-waste samples, under controlled (laboratory) (*n* = 9) and/or field conditions (*n* = 7), remains limited. Table 1, Table 2 and Table 3 summarise sample type and origin, characterisation before and after exposure, tested species/endpoints, and observed effects.

### 2.1. Laboratory Assays with Dismantled E-Waste and/or Derived Leachates

Evidence from laboratory studies on artificially produced e-waste leachates reveals a consistent pattern, with acute toxicity in aquatic invertebrates and algae scaling with leachate concentration, weathering treatment, and solid–liquid ratio (Table 1). For example, keyboards’ leachates induced 44 ± 24% mortality in the copepod *Nitocra spinipes* at 1:10 dilution, with UV-radiation pretreatment further enhancing toxicity—underscoring the role of photodegradation products in mobilising metals and organic chemical compounds [13]. Similarly, the algae *Scenedesmus vacuolatus* exposed to UV-weathered keyboards’ leachates exhibited EC_50_ values up to two orders of magnitude lower than those of non-weathered waste, with significant declines in fluorescence, cell density, and photosynthetic yield, indicating enhanced bioavailability of toxicants in aged leachates [14]. In zebrafish *Danio rerio*, borehole-water-leached PCB (Printed Circuit Board) and RAM (Random Access Memory) extracts (1:10 solid–liquid ratio) triggered neurotoxicity and oxidative stress, as evidenced by acetylcholinesterase inhibition, elevated catalase activity, glutathione depletion, and increased lipid peroxidation, highlighting the mixture effects of metals and flame retardants under field-simulated conditions [15]. Indeed, environmental weathering of e-waste materials facilitates the release of both organic (e.g., flame retardants) and inorganic (e.g., metals) chemical compounds over time [8,16].

Comparative leaching in distilled water versus artificial seawater from computer motherboards revealed that freshwater leachates released higher concentrations of Ni, Cu, and Zn, correlating with pronounced growth inhibition in multiple algae species (*Chlorococcum* sp., *Scenedesmus rubescens*, *Dunaliella tertiolecta*, *Tisochrysis lutea*), lysosomal membrane destabilisation in mussels (*Mytilus galloprovincialis*), and up to 6% micronuclei formation in human lymphocytes (further explored in Section 3). Conversely, seawater leachates, enriched in brominated organic chemical compounds, induced distinct genotoxicity and oxidative response mechanisms in the tested species, demonstrating how medium chemical composition and characteristics govern speciation and bioavailability of e-waste-related contaminants [17]. On the other hand, full devices (e-cigarettes, intact or disassembled) and potential leachates were found to alter the physiology of the aquatic plant *Lemna minor*, demonstrating how e-waste-derived products, particularly when disassembled, can impact primary producers in freshwater ecosystems [18].

Although these studies provided valuable knowledge on e-waste leachate toxicity, emphasising metal speciation as a primary driver of acute effects, with photochemically altered organic matter and ionic strength modulating sublethal oxidative and genotoxic pathways, they are limited when considering environmental settings. Tested dilution factors applied in laboratory studies (varying from 100% to 0.0001%) may only mimic those found in hotspots and neglect the co-occurrence of small-sized particles and leachates in the environment, which can interact in several ways [19]. This is of particular concern as e-waste processing and disposal sites may often be located near freshwater sources (rivers, lakes), which can, therefore, be more susceptible to direct contamination. Marine environments, while still vulnerable, may exhibit different toxicity profiles due to the potentially lower metal bioavailability under typical seawater conditions. Additionally, metals in seawater may form complexes or precipitates that alter their bioavailability, and these may precipitate and accumulate in sediments (as reviewed by Harmesa et al., 2024 [20]), thus posing greater threats to benthic organisms. This also applies to research that considers the whole e-waste/electronic equipment (as the example of e-cigarettes)—such studies would benefit from using environmentally representative area/volumetric loadings and spanning orders of magnitude to mimic different “doses”. Chemical characterisation of leachate concentrations in the medium would provide important insights into potential ecotoxicity drivers.

Additionally, ecotoxicity studies of both particles and leachates released from e-waste on terrestrial key organisms (e.g., plants, earthworms, and even rhizosphere/nitrogen-fixing microorganisms) remain, to our knowledge, poorly addressed (as discussed in Section 2.2). Soil compartments are less dynamic than aquatic ones, and the proximity of e-waste processing industrial activities or dumping sites may result in a combination of higher pollution levels, which can persist for longer. These factors may lead to progressively increasing concentrations of e-waste and related contaminants, underlining the importance of a renewed focus on ecotoxicity in terrestrial compartments.

**Table 1:** Laboratory ecotoxicity studies using e-waste and derived leachates, published between 2019 and 2025.

Tested Waste					Species	Endpoints	Observed Effects	Reference
Sample	Source	Processing	Exposure Medium	Concentration Gradient				
Keyboard housing plastic, cable coatings	Kjell, Stockholm, Sweden and unspecified waste processing facility, Norway	- Leaching in brackish water (with and without UV-radiation weathering)	- Brackish Water	- Up to 1/1 (*v*/*v*) leachate in exposure experiments, along with 1/3, 1/5 and 1/10	*Nitocra spinipes* (copepod)	- Survival	- E-waste plastic leachates induced some of the highest toxic effects across all tested polymers (44 ± 24% mortality at the lowest tested leachate concentration (1/10), up to 100% at 1/3 concentration)	[13]
Keyboard and unspecified microplastics	Unspecified e-waste sorting and recycling facility	- Leaching in saltwater (with or without UV-radiation weathering); leachates dried and resuspended	- GB medium	- 10 sequential 1:2 dilution steps	*Scenedesmus vacuolatus* (green microalgae)	- Chlorophyll α fluorescence - Photosynthetic activity - Cell density	- E-waste plastic leachates induced adverse effects in all parameters, with lower EC_50_ than those from other tested plastics (expressed in this study as effect values, or 1/EC_50_) - E-waste leachates induced lower photosynthetic yields (1.1, 1.3, 7.2 × 10^−1^, 1.1, 5.5 × 10^−1^, and 4.6 × 10^−1^ at 2 and 24 h, respectively) than procedural blanks (6.2 × 10^−3^, 2.6 × 10^−2^, undetected, 1.1 × 10^−2^, 5.7 × 10^−3,^ and 7.0 × 10^−3^, for 2 and 24 h, respectively)	[14]
Printed circuit boards and random access memory	University of Johannesburg, South Africa	- Leaching in borehole water for 4 weeks (solid-to-liquid ratio of 1:10)	- Borehole water	- 0, 25, 50, and 100% leachate concentration (*v*/*v*)	*Danio rerio* (zebrafish)	- Biochemical biomarkers	- Significant variation in acetylcholinesterase and catalase activity at different concentrations, reduced glutathione, glutathione S-transferase, malondialdehyde, and energy contents - Higher biomarker variations as leachate concentrations increase	[15]
Computer motherboards	Unspecified	- Computer motherboard leaching in distilled and artificial saltwater (35% salinity)	- Distilled water with BG-11 medium and Artificial saltwater with F/2 medium	- 100% (*v*/*v*) leachate concentration - 0.2, 1, 5, 10% (*v*/*v*) in the human lymphocyte test	*Chlorococcum* sp. (green algae), *Scenedesmus rubescens* (green algae), *Dunaliella tertiolecta* (green algae), *Tisochrysis lutea* (green algae), *Mytilus galloprovincialis* (Mediterranean mussel), *Homo sapiens* (Human)	- Growth -Cytotoxicity -Oxidative stress -Genotoxicity	- Significant growth inhibition (and cell number decrease) of *S. rubescens*, slowed growth of *T. Lutea* at 96 h. Significantly inhibited the growth of *D. tertiolecta* after 24 h. Non-significant growth inhibition in *Chlorococcum* sp - Significantly impaired mussel lysosome membrane stability at 24 and 96 h (as low as 65%), increased lipid peroxidation (over 10 times as high at 96 h), increased micronuclei frequency at 24 and 96 h, up to 5% (1% in controls) - Significant cytotoxicity in human lymphocytes (lower cytokinesis block proliferation index) and genotoxicity (micronuclei formation, up to 6% (3% in controls) on all concentrations.	[17]
E-cigarettes	Unspecified	- Three types of exposure: closed devices, disassembled devices and e-cigarette liquid	- Artificial freshwater	- 1 device per microcosm	*Lemna minor* (lesser duckweed)	- Growth	- Root growth slowed compared to controls and normal cigarette buts (biomass variation significantly reduced by approximately 40% compared to controls) - Open devices and e-cigarette liquid inhibit growth (approximately 40% reduction in biomass); no effect on chlorophyll contents - Closed devices induced weaker toxicity (approximately 30% reduction in the number of fronds relative to controls, compared to 40% open devices)	[18]

### 2.2. Laboratory Assays with Samples Collected from E-Waste Adjacent Environments

Compared to the laboratory-generated e-waste leachates, exposure to field-collected samples offers higher environmental realism as it considers natural weathering and complex contaminant mixtures. Bao et al. (2020) [21] and Igbo et al. (2022) [22] addressed the ecotoxicity of leachates and contaminated water (obtained from stream sediments and standing water contaminated with e-waste, respectively), aiming to simulate more environmentally relevant exposure scenarios. Bao et al. (2020) [21] demonstrated that leachates from sediments from e-waste dismantling sites in China significantly affected water flea *Daphnia magna* development (lower egg production, as low as 50%, and significant body length reductions, from 3.83 mm in control groups to as low as 3.38 mm) and shedding (increase in moulting frequency per adult during the 21-day exposure period up to 9.2, compared to 7.8 in control conditions), and altered expression of detoxification-related genes, underscoring acute toxicity potentially linked to metal and polybrominated diphenyl ethers, and polycyclic aromatic hydrocarbons detected in considerable concentrations in the medium (Table 2). Igbo et al. (2022) [22] provided direct evidence of toxicity from standing water collected near an e-waste facility in Nigeria in Atlantic killifish *Fundulus heteroclitus* embryos (impaired larval length and head sizes at all tested concentrations (0.0001% to 10% (*v*/*v*)) sample concentrations), connecting environmentally realistic exposure to organism-level effects. Lead, cadmium, chromium, and polycyclic aromatic hydrocarbons such as benzo(a)anthracene, chrysene, benzo(b)fluoranthene, benzo(k)fluoranthene, benzo(a)pyrene, and indeno(1,2,3)pyrene, were found to exceed safety limits delineated by US and Nigerian authorities in recovered samples. The effects reported in Bao et al. (2020) [21] and Igbo et al. (2022) [22] could not, however, be traced back to specific compounds. Complex pollutant speciation, sediment binding, and ageing processes may obscure which fraction of contaminants is bioactive, reducing mechanistic insight. Passive sampler deployment alongside biotic assays is infrequently used but could be valuable for monitoring freely dissolved toxicants.

Odnevall et al. (2023) [23] analysed the toxicity of airborne particulate matter collected near e-waste recycling yards on water flea *Daphnia magna* and in rainbow trout *Oncorhynchus mykiss* gill cells. The presence of such particles at a concentration of 74 mg/L induced 100% mortality in the water flea within 40 h, and decreased cell viability and increased oxidative stress (as inferred by the formation of reactive oxygen species) in fish gill cells, indicating potential aerosolised particulate-bound (eco)toxicity. The few tested concentrations (only two, which could be considered unrealistic compared to environmental settings) limit the ability to obtain a dose–response curve, and the use of tap water as a control might represent the presence of uncontrolled anthropogenic contaminants, which may cast doubt on the reliability of the results and limit ecotoxicity assessment. Furthermore, the use of particulate matter obtained directly from an e-waste facility on aquatic organisms may also limit the overall environmental relevance of the approach, considering the tested concentrations, given the dynamic nature of aquatic compartments and the dilution e-waste toxicants would suffer.

In the only study conducted on soil organisms, Nfor et al. (2022) [24] assessed the ecotoxicity of soil samples collected from informal e-waste recycling sites in Douala, Cameroon, where electronic components are burned and dismantled without protective measures (Table 2). Evidence showed metal contamination (Pb: 2850–12,400 mg kg^−1^; Cd: 15.2–89.6 mg kg^−1^; Cu: 1240–8760 mg kg^−1^) that exceeded international soil quality guidelines by 10–100-fold. Earthworms (*Alma nilotica*) incubated in these soils exhibited bioaccumulation factors of 0.8–2.3 for most metals, with tissue concentrations reaching lethal thresholds identified in laboratory studies. Earthworm reproduction was significantly impaired (50–80% reduction in cocoon production), and histopathological examination revealed tissue damage in digestive and reproductive organs. Importantly, the bioaccumulated metals in earthworm tissues pose secondary poisoning risks to predators, demonstrating trophic transfer pathways that are often overlooked in other studies. Beyond individual organism effects, the study documented shifts in soil microbial community structure, with decreased bacterial diversity and altered functional gene expression related to nutrient cycling.

Compared to aquatic systems, terrestrial environments remain an understudied field. The imbalance between aquatic versus terrestrial ecotoxicity assessment reflects several methodological and practical barriers. Soil matrices exhibit high complexity and special heterogeneity, characterised by varying organic content, pH, redox conditions, and particle size distributions that influence contaminant bioavailability. Unlike aqueous systems, where dissolved concentrations can be directly measured, soil-bound pollutants exist in multiple phases (dissolved porewater, exchangeable, organically bound, mineral-associated), requiring specialised extraction and fractionation procedures. Standard protocols like TCLP (Toxicity Characteristic Leaching Procedure) may not reflect actual bioavailability under field conditions. Notwithstanding, it is crucial to increase knowledge on terrestrial environments located near burning, dismantling, and e-waste burial practices, as they might present a higher permanence/accumulation of e-waste-related contaminants/pollutants over time, likely resulting in a higher likelihood of ecotoxicity and potential higher ecosystem risks.

### 2.3. Micro- and Macro-Biota Responses in Samples Collected from or Nearby E-Waste Facilities

Field investigations provide essential validation of laboratory toxicity assessments under realistic exposure conditions. The presence of metals, plastics, and persistent pollutants has been reported in e-waste processing sites and adjacent sites (e.g., Chai et al., 2020 [25]). Additionally, the incineration and chemical treatment of certain e-waste components can result in the volatilisation of organic compounds [26]. The release and fate of these e-waste-related pollutants locally (soil compartments) or in adjacent aquatic environments have the potential to affect local macro- and micro-biota and, consequently, impact ecosystem functionality, including agroecosystems as far as 1 km away from even inactive waste processing facilities [25]. However, the complexity of environmentally sourced samples and specimens may pose challenges to their full characterisation and to the replicability of conducted studies due to unforeseen and uncontrolled interactions between several different biotic and abiotic factors.

Soil and sediment microbiomes drive fundamental ecological processes, making their responses to e-waste contamination important indicators of ecosystem health and long-term sustainability [27,28]. Yet, field studies conducted so far revealed contrasting patterns of microbial vulnerability and adaptive responses that have important implications for contamination site management (Table 3). For example, Wu et al. (2019) [29] demonstrated that e-waste processing activities create distinct contamination gradients affecting microbial diversity. At burning sites, with elevated metal concentrations (419 ± 968 mg/kg), bacterial diversity declined substantially, with metals explaining 39.1% of community variation. Proximity to burning areas selected for thermophilic and acidophilic taxa, reflecting the harsh chemical and thermal conditions of informal processing. Critically, these effects persisted years after processing ceased (2013), indicating long-term ecosystem impairment.

Long et al. (2021) [30] found parallel declines, with overall microbial diversity decreased with metal contamination (mostly mercury). They also observed that the abundance of metal resistance genes increased proportionally. The correlation between the abundance of metal resistance genes and concentrations of specific metals was challenging to establish due to the dynamic and complex environmental setting, underscoring the importance of a more integrative approach that directly connects e-waste weathering/pollution dynamics and their ecotoxicological effects. Studies regarding the response of microbial communities to e-waste pollution could consider artificial (laboratory) approaches for controlled weathering and/or contamination of environments, and how observed endpoints compare to real-world scenarios. Applied to Long et al. (2021) [30], this would include the over-time evaluation of changes in microbial communities in soil spiked with real e-waste sources (under environmentally relevant pollution scenarios).

E-waste plastic particles can also induce changes to local microbial communities, given plastics’ potential to support the development of microbial microcosms on their surface, i.e., plastispheres [31]. Chai et al. (2020) [32] corroborated such a pathway with e-waste microplastics, resulting from burning activities, acting as vectors of hydrocarbon-degrading and pollutant-resistant bacteria. Incomplete combustion of e-waste might create reactive surface groups on released particles that enhance microbial colonisation [33,34], potentially accelerating contaminant biotransformation but also facilitating dispersal of resistance genes across environmental compartments.

**Table 2:** Laboratory ecotoxicity studies using environmental samples, published between 2019 and 2025.

Tested Waste					Species	Endpoints	Observed Effects	Reference
Sample	Source	Processing	Exposure Medium	Concentration Gradient				
Polluted stream sediment	Guiyu, People’s Republic of China (PRC)	- Leachates produced from retrieved sediments (metal and polybrominated diphenyl ether concentrations of 1657.14 µg/g and 7831.32 ng/g, respectively)	- M4 Medium	- 25%, 50%, and 100% (*v*/*v*) concentrations in exposure experiments	*Daphnia Magna* (zooplankton)	- Growth - Reproduction - Gene expression	- Increase in moulting frequency (7.8 to 9.2 within 21 days), days to first brood (5.3 to 9.1), days to first egg production (7.5 to 11.3) and decrease in number of eggs during first egg production (21.4 to 9.2), total eggs produced over 21 days (38.3 to 24.8, only at the highest concentration), and number of broods per female (3.3 to 1.9, only at 100% (*v*/*v*)) - Significant changes to gene expression, more prevalent at 100% (*v*/*v*) - Cytochrome P450 3 family and Vitellogenin gene inhibition at 100% (*v*/*v*); glutathione transferase gene inhibition.	[21]
Samples from standing body of water	E-waste dumping site, Alaba, Lagos, Nigeria	- Environmental samples with potential e-waste leachates (containing 0 0.2, 0.7 and 0.4 mg/L of lead, cadmium and chromium, respectively (exceeding US and Nigeria safety standards)	- Artificial Saltwater	- 10%, 1%, 0.1%, 0.01%, 0.001%, and 0.0001% sample concentrations	*Fundulus heteroclitus* (Atlantic killifish)	- Hatchability (survival) - Time until hatching - Growth and development	- Significantly lower body lengths in all concentrations compared to controls (as low as 2.47 ± 2.0 mm total length compared to 7.78 ± 0.05 mm) - Significantly different head sizes in all concentrations compared to controls (as low as 2.07 ± 0.07 mm compared to 4.35 ± 0.12 mm) - Significantly higher pupil size in all concentrations compared to controls (up to 2.18 ± 0.59 mm compared to 0.09 ± 0.02 mm) - Significant differences in all concentrations in yolk and eye iris sizes compared to control conditions (no clear correlation with concentrations)	[22]
E-waste recycling-generated particles	Unspecified recycling plant, northern Europe	- Filtered particles (majority under 100 nm (77%))—no processing after sampling	- Tap water (*D. magna*); L-15 medium with foetal bovine serum (*Oncorhynchu mykiss*)	- 2.3 to 7.5 mg/L (*O. mykiss*); 37 and 74 mg/L (*D. magna*).	*Daphnia Magna* (zooplankton), *O. mykiss* (rainbow trout)	- Cytotoxicity - Oxidative stress - Survival	- Decrease in *O. mykiss* gill cell viability only at the highest tested concentration - Increased formation of reactive oxygen species after 48 h of exposure compared to positive copper sulphate control - 100% mortality at the highest tested concentration after 40 h of incubation	[23]
Soil Samples	Douala, Cameroon	- Soil samples from 10 sampling sites, 8 related to informal e-waste processing—no processing after sampling	- Sampled soil; mean contamination factors (metal concentration in samples divided by those in non-e-waste soil) ranging between 2.42 and 569.79 for chromium, cobalt, cadmium, arsenic, nickel, mercury, zinc, lead, and copper		*Alma nilotica* (tropical earthworm)	- Metal ingestion and egestion	- General correlation between metal presence in soil samples and ingestion - Different metals ingested and egested at different rates - Mercury, copper, and zinc had slow egestion rates, whereas nickel had the most efficient egestion - Zinc represents the greatest contributor to ecological risk out of all metals	[24]

**Table 3:** Ecotoxicity assessment in micro- and macro-biota collected from e-waste processing sites and adjacent environments (literature survey between 2019 and 2025).

Tested Waste			Species	Endpoints	Observed Effects	Reference
Samples	Source	Tested Samples and Characteristics				
Soil	Guiyu, PRC	- Samples from farmlands, stream, and burning site in a waste processing region - Waste processing sites had higher pH (up to 6.41 ± 0.11) compared to farmland (5.78 ± 0.23), higher total carbon (up to 73.30 ± 33.75 g/Kg, compared to 20.15 ± 16.57 g/kg), and nitrogen (up to 3.77 ± 1.49 g/kg compared to 1.80 ± 0.83 g/kg) - Burning site soil had higher average metal concentration than the other two (419 ± 968 mg/kg compared to up to 86 ± 173 mg/Kg in farmland)	Microbial Community	- Community composition	- Presence of metals in soil contributed to reduction in microbial biodiversity (Pearson correlation analysis and factor analysis between genera and pollutants reveal positive correlation between metal concentrations and metal resistant genera in communities) - Larger proportions of thermophilic and acidophilic bacteria in e-waste burning site soil - Bacterial communities in incineration site presented lower biodiversity	[29]
Soil Microplastics	E-waste processing site, Guiyu, PRC	- Total of 45 polypropylene, polycarbonate and acrylonitrile butadiene styrene microplastics (5 of each type from each analysed plot)	Microbial Community	- Community composition adhered to soil microplastics	- Changes in bacterial communities with increased presence of hydrocarbon and pollutant-degrading bacteria	[32]
River Sediments	Unspecified river, Guangdong Province, PRC	- Eight sediment samples retrieved from 10 to 15 cm depth - Arsenic, Cadmium, Copper, Chromium, Mercury, Nickel, Lead, and Zinc generally exceeded local legal safety standards	Microbial Community (Firmicutes-dominated)	- Community composition - Prevalence of metal resistance genes	- Decrease in microbial diversity and increase in share of Firmicutes as contamination levels increased - Abundance of metal resistance genes increased as contamination levels increased	[30]
Organisms and River Sediments	Unspecified locations from river, Guiyu, PRC	- Individuals obtained from two e-waste polluted sites - Up to 7.01 μg/g, 1.01 mg/g, and 7.17 mg/g of cadmium, lead, and copper in river sediments in the most polluted sampling location - Liver metal burdens generally higher compared to control individuals	*Anabas testudineus* (climbing perch)	- Transcriptome changes related to stress response and DNA repair	- Different metabolic responses to different environmental contamination - Downregulation of redox, hypoxia, and protein transcription and translation-related genes - Upregulation of redox and apoptotic process-mediating genes - Altered DNA repair responses, less intensive in the specimens sampled from the more polluted site	[35]
Organisms	Contaminated environments, Guiyu, PRC	- Individuals obtained from three e-waste polluted sites - Metal concentrations in the source environments unspecified - Liver metal burdens generally higher compared to control individuals	*A. testunideus* (climbing perch)	- Gut microbiota changes	- Site-specific beta diversity, and correlation between specific metals and bacteria, namely *Cetobacterium somerae* and *Clostridium colicanis* with manganese and lead, respectively	[36]
Organisms	Contaminated Environments, Guangdong Province, PR	- 138 adult individuals obtained in twelve sites, two related to e-waste processing	*Alcedo atthis* (common kingfisher bird)	PBDE (Polybrominated diphenyl ethers), AHFR (Alternative Halogenated Flame Retardants), and PCB burdens in muscle tissue	- Up to 3 × 10^5^ ng/g and 1.5 × 10^6^ ng/g of PBDEs and PCBs in lipid mass of kingfishers sampled near e-waste processing sites - Significant relationships between contaminant burden and both distance to e-waste processing site and human population density	[37]
Organisms	Contaminated environments, Longtang Town, PRC	- Individuals obtained from an e-waste-contaminated pond - Short- and medium-chained chlorinated paraffin concentration in the environment unspecified	*Enhydris chinensis* (Chinese water snake), *Cyprinus carpio* (Eurasian carp), *Macrobrachium nipponense* (freshwater shrimp), *Anaurornis phoenicurus* (white breasted waterhen)	- Biomagnification - Maternal transfer	- Short- and medium-chain chlorinated paraffins with higher accumulation rates in water snake eggs than in the specimen (37–73% accumulated in the eggs, 27% of the weight of the water snake), indicating maternal transfer - Potential occurrence of biomagnification in the fish-water snake food chain	[38]

Macrofauna collected from e-waste-contaminated sites can provide key insights into the health of the local ecosystem as well as the spread of e-waste contamination from a hotspot. Zhang et al. (2019) [35] conducted the most comprehensive organism-level assessments using the climbing perch *Anabas testudineus*, a mud-dwelling species with high exposure potential. Transcriptomic analysis of liver, gill, and kidney tissues revealed dose-dependent responses: moderately contaminated sites induced few stress responses (redox and apoptosis genes), while heavily contaminated sites showed impaired DNA repair—suggesting threshold effects where adaptive capacity becomes overwhelmed. In another study, parallel shifts in gut microbiota composition (site-specific β-diversity) were found to indicate systemic physiological disruption extending beyond direct toxicity [36]. Given its mud-burrowing and crawling behaviour, and its role in the aquatic food web, this species could be especially exposed to denser e-waste-related pollutants, such as metals and denser plastics that become mixed into sediments [39]. Changes to the gut microbial communities could have implications for fish gut metabolic function and resistance to pathogenic microbes [40,41], whereas tissue bioaccumulation of metals and/or organic e-waste contaminants might have implications for trophic transfer and eventual magnification [42].

For example, Peng et al. (2019) [37] documented high bioaccumulation of polybrominated diphenyl ethers in kingfishers near e-waste sites (up to 1000-fold higher than at reference sites), with concentrations correlating with human population density—directly linking consumer electronics use to wildlife contamination. Exposure to these compounds was also estimated to pose ecological risk to the surveyed species’ reproduction through the calculation of hazard quotients. In the study described in Guan et al. (2020) [38], bioaccumulation and transfer of short- and medium-chain chlorinated paraffins (CPs) in an e-waste-contaminated pond and surrounding areas were assessed, sampling water snake (*Enhydris chinensis*), carp (*Cyprinus carpio*), shrimp (*Macrobrachium nipponense*), and waterhen (*Anaurornis phoenicurus*). High concentrations of short-chain CP—up to 250 µg/g lipid in snake muscle—far exceeded levels reported in non-e-waste regions and demonstrated maternal transference with 37–73% of maternal burden, establishing multigenerational exposure pathways often overlooked in risk assessment. Medium-chain CPs (C_14_–C_15_) exhibited lower but homogeneous tissue concentrations in all species. Results suggest that both low-molecular-weight congeners preferentially bioaccumulate in eggs and transfer across trophic levels. Biomagnification factors also differed between the fish–water snake and fish–waterbird food chains, suggesting potential for biomagnification mainly in the former. The relatively low sample sizes (up to 7 sampled individuals per species) in Guan et al. (2020) [38] may limit statistical power and generalisability, and the lack of concurrent environmental measurements of target contaminants in water and sediment prevents calculations of bioaccumulation factors and dose–response relationships.

### 2.4. E-Waste Ecotoxicity: Key Research Gaps and Solutions

**Figure 1:**
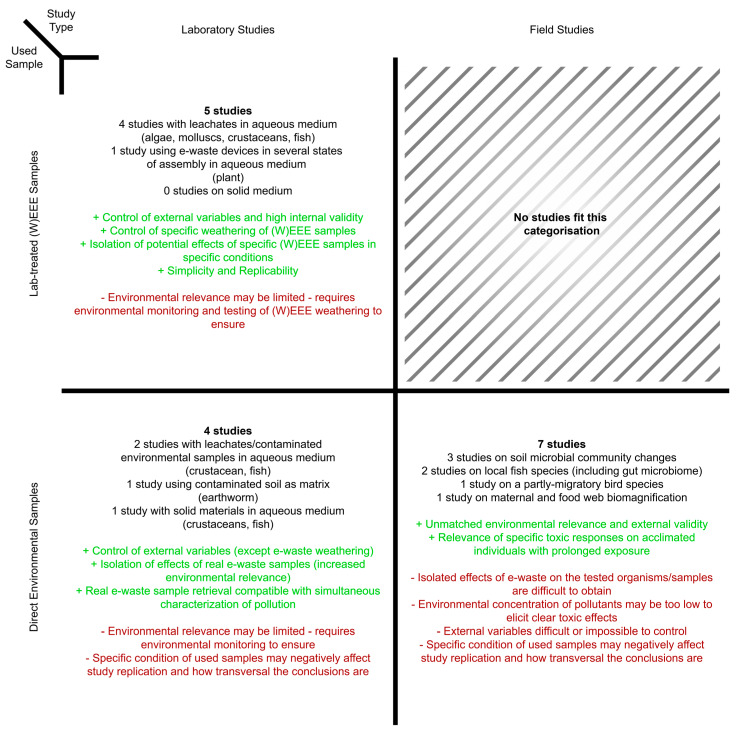
Overview and general advantages (in green) and disadvantages (in red) of the studies surveyed in this critical review, which fit into three main categories (laboratory studies using laboratory-treated (W)EEE samples, laboratory studies using environmental samples, and field studies (with environmental samples)).

The studies discussed above demonstrate that, under specific conditions, samples from e-waste and nearby environments can cause both acute and chronic ecotoxicity. Despite the value of the collected data and knowledge, some procedural challenges remain, as summarised in Figure 1.

Laboratory studies face several limitations that reduce the comparability of data/results across studies. These include variability in sample characteristics (form factors, formulation of the used waste, weathering state of the used samples, different constituents), differences in exposure routes among tested organisms, the diverse range of organisms examined. In addition, the environmental relevance of the tested concentrations of e-waste and related toxicants is questionable, with an overall lack of e-waste samples characterisation in relevant settings (e.g., compartments in the proximity of current and former e-waste processing industries)—which can contribute to a lack of environmental relevance. Furthermore, most laboratory studies with aquatic species focused on testing e-waste artificially derived leachates, which may not represent the most immediate threat from e-waste given aquatic compartments’ higher dynamic nature and potential for spread and dilution of contaminants. A relative minority of lab studies have focused on e-waste in solid/particulate form.

Future laboratory-scale studies would benefit from uniformized methodologies to improve the comparability and integration of collected data. Specifically, future research should use (i) a set of representative model organisms; (ii) renew focus on soil compartments whose relative proximity to industry often leads to higher contamination; (iii) select, prepare, and test different forms of e-waste sources, fragments, particles, and leachates using consistent procedures; and (iv) apply harmonised methods for chemical characterisation of toxicants. An increased control over such variables could contribute to isolate the potential (eco)toxic effects of e-waste. Using Nfor et al. (2022) [24] as an example, a ground-up laboratory-scale study (i.e., using characterised e-waste toxicants) could help elucidate the potential effects of increased concentrations of the toxicants and potential changes to the properties of the soil. In addition, given the current low number of studies in terrestrial compartments compared to aquatic compartments, studies in terrestrial compartments should be regarded as a future research priority. On the other hand, an assessment in relevant environments could elucidate longer-term impacts under extant scenarios. Moreover, this harmonisation of methodologies could be essential for the development and validation of new approach methodologies (NAMs), allowing for more mechanistic, high-throughput, and animal-reduced testing strategies. Some studies discussed in previous sections (e.g., Odnevall et al., 2023 [23]) use such approaches, particularly in vitro systems (cell lines/derived cell models). However, given the chemical complexity and heterogeneity of e-wastes, which can include a mixture of different stressors with differing impacts on various biological systems [43], it remains crucial to conduct conventional ecotoxicological assessments alongside NAMs, probing e-waste toxicity across multiple levels of the adverse outcome pathways (e.g., organ, organismal, and populational-level events). In this sense, a robust and harmonised set of e-waste (eco)toxicity studies can provide a solid, environmentally relevant basis for interpreting and extrapolating results obtained through these approaches and for validating NAMs’ fitness for ecotoxicity assessments. For instance, while Odnevall et al.’s (2023) [23] use of *O. mykiss* gill cells presents valuable insights into the potential for e-waste to induce oxidative stress on a key system of the rainbow trout, it is also essential to understand how such cellular responses may relate to, or trigger, eventual organismal or population-level effects.

Expanding environmental monitoring around e-waste processing sites and relevant e-waste reservoirs, such as uncontrolled dumps and brownfields, along with thorough characterisation of recovered waste, is also essential to clarify e-waste’s role in overall pollution. Although such detailed workflows can be time-consuming, they are essential. Data from Zhang et al. (2021) [44] reported 14,200 particles/153 mg per kg of soil in the vicinity of e-waste processing industries. These concentrations can be used as a realistic mid- and high-range for future toxicity tests, enabling studies to assess both relevant exposure levels and dose-dependent toxicity. Such data could also be crucial to incentivise public policy and regulatory action, and to address legislative gaps found at national and international levels [45,46,47].

**Figure 2:**
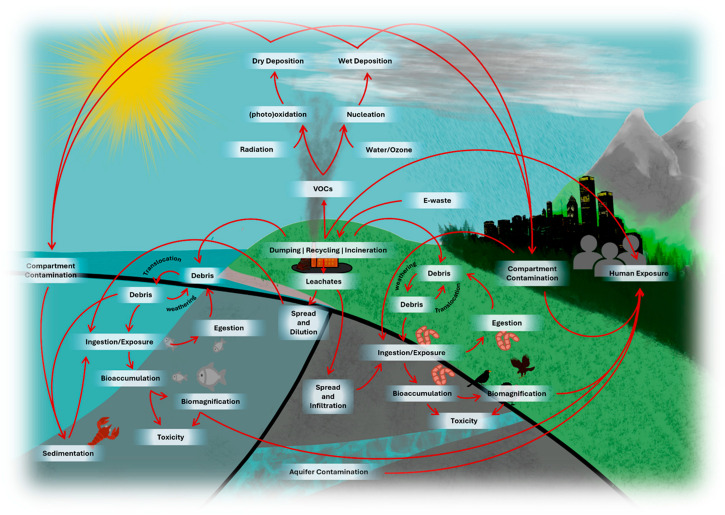
E-waste constituent release, environmental contamination, and main biological exposure routes.

Field studies face similar challenges in characterising pollution. The complexity of field conditions, including diverse physicochemical transformations, environmental dispersion pathways, and multiple routes for e-waste contamination (e.g., Fetisov et al., 2023, [48] Shaaban et al., 2024 [49]), as shown in Figure 2, can obscure potential toxic effects.

The original setting of the e-waste may also play a role, as the release, transformation, transport, and bioavailability of e-waste-derived pollutants may differ depending on whether they originate from active e-waste processing activities (formal or informal), dumping sites, urban areas, or derelict locations/brownfields (e.g., abandoned e-waste sites, as described in Zhang et al. (2021) [44]). However, these factors require further study. Moreover, organisms’ prolonged exposure and possible adaptation to e-waste pollutants and unrelated contaminants may further hide (eco)toxic responses. Therefore, laboratory and field studies play complementary roles in understanding e-waste ecotoxicity, each with distinct advantages and challenges (as outlined in Figure 1). Laboratory studies enable isolation and assessment of specific e-waste toxicants, while field studies show how acclimatised populations respond to realistic long-term exposure scenarios.

Considering long-term exposures, the trophic transfer and parental transfer of e-waste contaminants, particularly metals and microplastics that can carry additional pollutants, are inadequately covered. Guan et al. (2020) [38] examined chlorinated paraffins, but similar research on metals and plastics is lacking in recent research. In addition, multi-generational studies, including biomagnification and effects on reproduction, behaviour, and immunity, have not been explored.

Finally, the (eco)toxicity of e-waste treatment byproducts and effluents remains almost entirely unexplored. Arya et al. (2021) [50] is the only study (to our knowledge) testing chemical leachates’ filtrates (resulting from a chemical leaching process of various e-waste materials, with and without epoxy coating treated with hydrochloric acid). Post-treatment filtrates were characterised for the presence of metals and tested according to the US EPA method #1311 [51]. Results from this test classified the NaOH-treated waste filtrate residues as hazardous, considering the amount of copper present exceeded the defined threshold. Those not submitted to the treatment were also classified as hazardous due to the amount of lead. No pH values for the generated effluents used in this test were provided; this data could further inform the environmental impact of the procedure. As it stands, the only assessment of (potential) toxicity was made through this test, which is based merely on the concentrations of specific compounds, providing limited insights into potential interactions between them and materials from the associated matrix. Further, and as previously mentioned, studies using representative organisms would contribute to a better understanding of the (eco)toxicological impacts of these procedures.

## 3. Human Exposure and Toxicity

The persistence and spread of e-waste hazardous compounds, coupled with industrial e-waste processing, may also result in human exposure, particularly among those closest to or involved in processing activities (especially in informal settings) [52]. A variety of studies have also been conducted among human populations (Table 4), focusing mostly on occupational e-waste workers and populations in geographic proximity to e-waste-related industries.

Several effects have been reported, such as metabolization and potential maternal transfer of OPFR (Organophosphate Flame Retardants) and PBDE metabolites (e.g., Bai et al., 2019 [64]; Matovu et al., 2019 [65]), inflammatory responses (e.g., Chen et al., 2021 [63]), altered immune and antimicrobial response (e.g., Chen et al., 2021 [63]; Zhang et al., 2020 [58]), and DNA damage/alterations (e.g., Alabi et al., 2020 [54]; Zeng et al., 2022 [62]). These studies demonstrate the potential negative effects of e-waste exposure on the health of populations, both those directly involved in e-waste management and those adjacent to such industrial activities. Additionally, it is estimated that human populations settled near or directly involved in e-waste processing industries face higher risks of congenital malformations and cancer, according to risk assessment studies such as Dai et al., 2025 [66] and Matovu et al., 2019 [65]. This highlights the need for better e-waste management and territory organisation policies, duly informed by contamination assessment and (eco)toxicity studies.

However, human studies, in general, may suffer from limitations related to sample sizes and varying degrees of exposure to contaminants, which can contribute to data biases, result variability, the presence of confounding variables, data inconsistencies, and reduced internal validity of the procedures, ultimately resulting in comparability challenges. To that end, smaller-scale confirmatory studies would be needed, using model organisms under controlled conditions, as discussed in previous sections. Going forward, these should also consider including an assessment of contamination of the environment concurrent with biological sample collection, as well as a broader exploration of biomarkers regarding, for example, inflammatory response and DNA damage.

The study of how e-waste-related toxicants affect human cell lines can also help clarify potential health risks. For example, Kalamaras et al. (2021) [17] (described in Table 1) investigated the effects of e-waste leachates on human lymphocytes using cytokinesis block micronucleus assays, with results indicating cytotoxic and genotoxic effects. Significant differences from the control were observed as leachate concentrations increased (above 1%, including), with micronuclei formation rising up to two times more frequently. These effects may be triggered by exposure to some metals and persistent organic pollutants, potentially reflecting broader DNA damage (such as chromosomal damage or mutations). On the other hand, Shi et al. (2021) [67] found limited toxicity when human cell lines (keratinocyte, colon, bone osteosarcoma, and mesenchymal stem cells) were exposed to e-waste plastics, maintaining cell viability above 90%, despite the metals in the plastics exceeding safety standards (e.g., European Parliament and Council Directive 2011/65 RoHS 2 [68]). Nevertheless, this study’s focus on using e-waste materials and the specific exposure scenario (directly seeding cells onto plastic surfaces) may have resulted in lower leaching of toxic compounds. Longer-term research is necessary to understand how direct physical contact with e-waste plastics, without weathering or extraction of contaminants, might induce toxicity in human cells. Such studies would also enhance the relevance of findings, considering the widespread use of electronic devices and the occupational exposure to e-waste. In this context, testing for DNA damage (linked to potential carcinogenicity and reproductive or developmental toxicity) and immune system impairment is crucial for assessing long-term public health impacts and guiding policy decisions.

## 4. In Silico Tools for (Eco)Toxicity Assessment

Given the challenges inherent in conducting ecotoxicity studies on complex matrices, such as e-waste, in silico tools for ecotoxicity assessments, including consensus models and Life Cycle Assessment (LCA) approaches, are gaining momentum. LCA, with its cradle-to-grave approach, offers a broader perspective by assessing the impacts of material and process components throughout their life cycle. This whole system of thinking contrasts with the focus on individual aspects in many ecotoxicity studies, and LCA can provide valuable information for decision-making.

Singh et al. (2020) [69], for example, used LCA to estimate the potential toxicity of e-waste plastics from mobile phones, selected from a pool of popular obsolete devices from a recycling business. After disassembly, the contents of metals, tetrabromobisphenol A, hexabromocyclodecane, and other persistent organic pollutants were determined. A Life Cycle Impact Assessment (LCIA) was subsequently conducted using the USEtox LCIA model to estimate the risk posed by the analysed waste. The results obtained in this study indicate the potential toxicity of mercury, lead, chromium, antimony, and bromide, with mercury posing the highest overall cancer risk and chromium posing the highest ecotoxicity risk. While these results focus mainly on a subsect of e-waste (i.e., e-waste-derived housing plastics, rather than complex e-waste with several different constituents, i.e., a full device), providing valuable information regarding the potential impact of these wastes, they fail to inform the extent of the impact on key species in the same way the previously discussed ecotoxicity studies did, instead offering a more general perspective.

Furthermore, while LCA approaches are increasingly popular and important from a public decision-making standpoint [70,71], the variety of purpose, composition, expected lifetimes, and modelling choices achieved by the researchers all lead to variability in results, and represent challenges to the comparability of these studies [72]. Moreover, such studies rely on previously built libraries and consensus models (such as USEtox) that allow for estimations of the impacts of the used components, processes, and logistics, whose robustness relies on the availability and wealth of data, often derived from a limited pool of endpoints measured exclusively under laboratory conditions, resulting in inconsistent environmental relevance. Subjective choices of different factors in study design can also contribute to variability in results, as does the choice of library and model [73]. Furthermore, the potential mix of experimental and modelled (i.e., Quantitative Structure-Activity Relationship) datasets informing the LCIA calculation may also increase the uncertainty of the results. These common problems are further compounded when considering e-waste, as a whole. Impact in LCIA is calculated through the sum of the impacts of each single element, and thus, the interaction between different constituents becomes difficult to determine.

This potential for variability runs contrary to the LCA’s purpose as an informative tool for policy and industry; however, the role and use of these tools has increased over the years due to an increased regulatory dependence on results from such models. For example, calls for proposals under European Union plans for research programme funding have increasingly required or underlined the need for such LCAs, but not necessarily incentivizing proper (eco)toxicological and trials of different product components in conjunction [74,75]. However, the lack of a thorough understanding of the effects and interactions of different components and subsequent effects could foster a false sense of security brought about by the theoretical result of the LCAs, which is in itself susceptible to subjective decisions. It is thus important that further calls for funding become more aware of the implications of LCA studies and push for a more robust understanding of e-waste, its toxicants, and their potential interactions by incentivising the execution of ecotoxicological assessments alongside them. Furthermore, just like with ecotoxicological studies, LCAs would also benefit from efforts towards harmonisation of methodologies. Initiatives have been conducted in this direction—for example, the European Commission’s Product Environmental Footprint Category Rules [76]. However, the impact category definition is limiting, from an ecotoxicity perspective—the only ecotoxicity impact category contributing to the overall calculated impact is “freshwater ecotoxicity”, accounting for 0.1% of the total calculated impacts (human toxicity categories, on the other hand, account for 23.2%). In summary, beyond establishing stronger data foundations (such as studies on e-waste (eco)toxicity), it is necessary to assess the potential fates and processing methods of e-waste (e.g., formal, informal, dumping), model the transport and distribution of e-waste constituents in relevant environments, and investigate and model the toxicity of various representative toxicant mixtures in e-waste to complement current approaches. This latter topic involves defining chemically representative mixtures of toxicants and studying their mechanisms of toxicity. Subsequently, potential interactions among these toxicants could be examined in predictive models using concentration addition or independent action for particular mechanisms (e.g., neurotoxicity, genotoxicity), subject to experimental validation [77,78].

## 5. Conclusions and Recommendations

The escalating volumes of e-waste and its pervasive mismanagement, including hazardous processing practices and transboundary movements, pose a significant threat to both human health and the integrity of ecosystems. E-waste’s inherent material complexity imposes direct ecotoxicological studies to accurately assess the risks posed by its diverse constituents and their interactions, as single-exposure assessments fail to capture the reality of complex mixtures.

Over the past five years, several studies have been published exploring the (eco)toxicity of e-waste, using either e-waste directly, leachates obtained from e-waste, and requires environment, or biological samples from areas associated with e-waste processing. Most of the studies conducted during this timeframe showcased the diversity of the toxic effects of e-waste, its constituents, and released pollutants on key species, including primary producers, key intermediaries, and nutrient cyclers, as well as human exposure and the transfer of toxic compounds. While the environmental relevance of the tested conditions is challenging to assess, the adverse effects induced by (co-)exposure to e-waste and related contaminants remain a special concern, given the potential for bioaccumulation and biomagnification, which poses a long-term threat to ecosystems. The material and form-factor complexity of e-waste may also affect the replicability and comparability of studies. In this context, the definition of representative e-waste samples could help overcome some of these challenges; however, such materials would then need subsequent updates, given the potential changes to the composition of different subsections of EEE, e.g., the progressive replacement of PBDEs in electronics by AHFRs [79]. On the other hand, studies using environmental samples, and thus with potential for higher environmental relevance should not be disregarded.

E-waste treatment options would also benefit from a better understanding of the ecotoxicological impacts resulting from their activity. Strategies for the recovery of precious metal components, as well as for the decontamination, recycling, or disposal of plastic components, may incur additional environmental hazards, through the emission/release of toxic compounds from the waste materials themselves during processing, or the presence of hazardous chemicals in effluents from hydrometallurgical or solvent extraction approaches, for example. In the past years, many studies have been published regarding the impacts of different processing strategies for different types of e-waste and their components following an LC(I)A methodology, whereas ecotoxicological studies using residues generated from such processes lagged. However, given that the LCA model has disadvantages that could affect the relevance of the obtained results, further ecotoxicological studies need to be conducted to supplement this knowledge and contribute to more robust LCA models and results. However, the latter have not been keeping up with the former in this review’s target timeframe: as an example, at the time of writing, using the platform Scopus and the query “e-waste AND ecotoxicity OR e-waste AND LCA” for article title, abstract, and keywords delivers a total of 68 research articles; using the query “e-waste AND LCA” delivers the self-same 68 documents (mostly related to e-waste processing, rather than the impact of the waste itself). “e-waste AND ecotoxicity”, on the other hand, yields 48 research articles. This suggests LCA-related research also seems to outpace ecotoxicity assays, though mainly in the topic of e-waste processing.

Moving forward, an increase in studies practically testing the ecotoxicological effects of different e-wastes, constituents, and released pollutants will become essential to better understand their exposure pathways and potential ecotoxicological impacts of such exposures, safeguarding the health and safety of both populations and ecosystems at large, and ensuring the accuracy and reliability of developed NAMs and risk assessment models.

## Figures and Tables

**Table 4:** Human exposure and toxicity studies using e-waste sources published between 2019 and 2025.

Location	Population	Assessed Toxicity	Observed Effects	Reference
Ohio, USA	Occupational	Mercury exposure	Increased urine mercury levels, symptoms consistent with mercury toxicity	[53]
Lagos, Nigeria	Occupational	Metal exposure, DNA damage	Increased blood metal levels, increased micronucleus formation in buccal cells	[54]
Taizhou, PRC	Adjacent residents	Metal exposure	Biomarkers indicative of fibrosis risk	[55]
Guiyu, PRC	Adjacent residents	Lead and PAH (polycyclic aromatic hydrocarbon) exposure	Blood lead levels and urine PAH metabolite levels, biomarkers associated with inflammation	[56]
Vietnam	Occupational	Persistent organic pollutants in blood	Higher contamination levels in e-waste processing workers	[57]
Guiyu, PRC	Adjacent residents	Lead exposure	Blood lead levels correlated with peripheral monocyte percentage	[58]
Madina Zongo, Ghana	Occupational	Heart rate variability	Particulate matter correlated with irregular cardiac function	[59]
Not Described	Occupational	Hepatotoxicity biomarkers	Reduction in glutathione levels	[60]
Payatas, Philippines	Occupational	Binucleated cell frequency	Frequency of binucleated cells higher in e-waste workers	[61]
Guiyu and Haojiang, PRC	Adjacent residents	Endocrine disruption, DNA methylation, developmental disruption	Negative relationship between particulate matter exposure and birth head circumference	[62]
Guiyu and Haojiang, PRC	Adjacent residents	Inflammation and immune response	Higher PAH levels correlated with inflammation in the intestinal epithelium and activated immune response	[63]

## Data Availability

No new data were created or analyzed in this study. Data sharing is not applicable to this article.

## Abbreviations

The following abbreviations are used in this manuscript:

EEE	Electrical and Electronic Equipment
WEEE	Waste Electrical and Electronic Equipment
PCB	Printed Circuit Board
RAM	Random Access Memory
TCLP	Toxicity Characteristic Leaching Procedure
CPs	Chlorinated Paraffins
PBDEs	Polybrominated Diphenyl Ethers
AHFRs	Alternative Halogenated Flame Retardants
NAMs	New Approach Methodologies
PAHs	Polycyclic Aromatic Hydrocarbons
OPFRs	Organophosphate Flame Retardants
LCA	Life Cycle Assessment
LCIA	Life Cycle Impact Assessment
